# Editorial: Mechanisms of Vessel Development: From a Primitive Draft to a Mature Vasculature

**DOI:** 10.3389/fphys.2021.725531

**Published:** 2021-07-16

**Authors:** Sara Petrillo, Anna Rita Cantelmo, Tullio Genova, Luca Munaron

**Affiliations:** ^1^Department of Molecular Biotechnology and Health Sciences, Molecular Biotechnology Center (MBC), University of Turin, Turin, Italy; ^2^University Lille, Inserm, U1003—PHYCEL—Physiologie Cellulaire, Lille, France; ^3^Department of Life Sciences and Systems Biology, University of Turin, Turin, Italy

**Keywords:** endothelial cell heterogeneity, vascular remodeling, vascular maturation, blood flow, vessel enlargement

## Tissue-Specific Endothelial Cell Heterogeneity

Vascular heterogeneity has become a hot topic over the past 5 years. New and advanced molecular techniques, such as single-cell sequencing (scRNA-seq), have contributed to answer fundamental questions on how the different phenotypes adopted by endothelial cells (ECs) in the organ in which they reside shape the architecture and function of the vasculature. Understanding tissue-specific EC heterogeneity is crucial for targeted therapies which aim to deliver therapeutic agents to specific organs. Although vessel heterogeneity is defined by signals from the tissue microenvironment, the picture is much more complex than anticipated as gene expression patterns have been found shared among different tissue types. In this regard, Fleischer et al. provided an extensive overview of the structural and functional properties of ECs among tissue-specific vascular beds, highlighting the contribution of EC plasticity to pathological angiogenesis.

Endocrine signals ensure a peculiar vascular niche that is directly involved in the temporal control of hormone secretion and entry into the bloodstream (Stucker et al.). The complex interplay between endothelial and endocrine cells underlies the endocrine function. Consequently, vascular alterations may lead to endocrine disorders. Among the endocrine organs, the adipose tissue and its dense vascular network, have gained attention. Herold and Kalucka highlighted the role of the vascular system in the regulation of adipocyte function and adipogenesis. Interestingly, obesity is characterized by an angiogenesis-dependent rapid expansion of the adipose tissue: such dysfunctional environment is sustained by alterations in the paracrine signaling that governs the crosstalk between adipocytes and ECs. This evidence suggests that angiogenesis may be targeted for therapeutic purpose to ameliorate the complications and comorbidities associated to obesity.

Alfaidy et al. gave us another example of how a deep comprehension of the mechanisms involved in tissue-specific angiogenesis can provide novel tools for the treatment of human diseases. They discussed in detail the contribution of endocrine gland derived-vascular endothelial growth factor (EG-VEGF) in placental vascularization during pregnancy. Importantly, EG-VEGF is a member of the prokineticin family and a placental pro-angiogenic factor that has been proposed as a biomarker in preeclampsia, one of the most threatening pregnancy disorders.

Several zebrafish and mouse model systems contributed to the understanding of the complex brain vessel patterning. Brain ECs show many peculiarities, especially in light of their interaction with astrocytes and neurons within the neurovascular unit. Although significant progress has so far been made in characterizing brain endothelium, yet much is unknown regarding how brain vessels organize. Gupta et al. comprehensively summarized all the established, new and emerging concepts in brain vascular development. A crucial issue in the study of brain ECs concerns the difficulty to obtain reliable *in vitro* models that mimic the physiological complexity of the blood brain barrier. Indeed, mammalian primary brain ECs easily lose their barrier function upon culture. A rigorous phenotypic characterization is therefore fundamental to assess the value of EC culture models. Lu et al. addressed this point in depth and clearly demonstrated that human pluripotent stem cells (hPSCs)-derived brain microvascular ECs (iBMEC) are not phenotypically comparable to primary human brain microvascular cells (BMECs), but rather display the features of an epithelial barrier. The need for single-cell level analysis as scRNA-seq for proper characterization of hPSCs-derived ECs also emerges from the work of Helle et al. Indeed, they showed that despite hPSC-derived ECs can adequately respond to flow-induced shear stress, they nevertheless retain an immature nature and tendency to transdifferentiate, making them different from primary ECs.

Vascular alterations in the central nervous system (CNS) are very detrimental and often not compatible with life. Moreover, vessels modifications are involved in the pathophysiology of glioblastoma (GB), one of the most aggressive CNS tumors in adults. The morphological and functional features of the brain tumor vasculature in GB have been extensively described by Guyon et al. Interestingly, the authors provided an excursus of useful tools that allow reproducing and studying tumor-stroma interactions and can be exploited for therapeutic applications. Among them, tissue-engineered blood vessels and 3D co-culture models represent an attractive alternative to animal models and may be used in drug-screening to identify improved therapies.

## Cell Dynamics in Shaping Vascular Remodeling

Newly formed vessels are immature, and they must undergo remodeling to achieve a functional network. Microvascular remodeling is a dynamic process, which reflects the dynamic nature of blood flow. Santamaría et al. provided a comprehensive description of the mechanisms that drive remodeling, from the crucial role of flow forces to the chemical signals and mechanical cues involved. To reach an adequate vascular patterning, ECs integrate mechanical and chemical signals and translate them into functional behaviors. Interestingly, Vion et al. showed that vascular endothelial growth factor receptor 2 (VEGFR2) is a sensor able to integrate chemical and mechanical information and control cell shape.

Importantly, blood flow is a master regulator of vascular remodeling not only by promoting vessel pruning or splitting, but also by regulating vessel enlargement. The critical role played by vessel enlargement during both development and adulthood has been discussed by Gifre-Renom and Jones, who highlighted the mechanisms involved in the process, yet pointing out the need for further studies that may open new translational opportunities.

A deeper understanding of the molecular factors which dynamically shape the transcriptional landscape of ECs under different environmental and developmental conditions, is also fundamental. In the last years, several molecular players involved in vascular maturation and remodeling have been identified. In their review, Doronzo et al. summarized the latest advances on the transcription factor EB (TFEB), which acts as a hub in vessel remodeling and maturation by activating specific genetic programs currently under investigation.

Finally, an interesting perspective and promising strategy for therapeutic angiogenesis comes from emerging studies on resident endothelial progenitors' dynamics. Endothelial colony forming cells (ECFCs) are endothelial progenitors that are mobilized in the peripheral blood to maintain endothelial homeostasis and/or facilitate neovascularization in diseased tissue. Moccia et al. addressed this topic, and reported the most advanced strategies enrolled to boost the regenerative potential of ECFCs, including intracellular calcium mobilization upon stimulation with light.

## Author Contributions

All authors listed have made a substantial, direct and intellectual contribution to the work, and approved it for publication.

## Conflict of Interest

The authors declare that the research was conducted in the absence of any commercial or financial relationships that could be construed as a potential conflict of interest.

